# Prevalence of *Schistosoma mansoni* infection among children in Ethiopia: a systematic review and meta-analysis

**DOI:** 10.1186/s40794-021-00156-0

**Published:** 2021-12-01

**Authors:** Habtye Bisetegn, Tegegne Eshetu, Yonas Erkihun

**Affiliations:** 1 College of Medicine and Health Sciences, Department of Medical Laboratory Sciences, Wollo University, Dessie, Ethiopia; 2 College of Medicine and Health Sciences, School of Biomedical and Laboratory Sciences, Department of Medical Parasitology, University of Gondar, Gondar, Ethiopia

**Keywords:** Schistosomiasis, *Schistosoma mansoni*, Children and Ethiopia

## Abstract

**Background:**

Schistosomiasis is a neglected tropical disease caused by mainly *Schistosoma mansoni* and *Schistosoma hematobium*. The disease is very common in Africa including Ethiopia. *Schistosoma mansoni* is a major public health problem in Ethiopia especially among children. This review is aimed to indicate the prevalence of *Schistosoma mansoni* among children at the national and regional levels.

**Methods and material:**

The PRISMA guidelines were followed. An electronic search of PubMed, Google Scholar, Web of Science, Scopus, MEDLINE, and Google search were carried out using key terms. Articles published from the proceeding of professional associations such as the Ethiopian medical laboratory association, the Ethiopian public health association, and annual national research conferences were also searched to find additional eligible studies. Data were extracted independently by two investigators, and cross-checked by a third reviewer. The quality of included studies was assessed using JBI quality assessment criteria. Data were extracted using Microsoft excel and finally analyzed using STATA version 12. The pooled prevalence was done using a random-effects model.

**Result:**

Overall 49 studies involving 20,493 children (10,572 male and 9, 921 females) were included in this meta-analysis. The pooled prevalence of *Schistosoma mansoni* infection was 37.13% (95%CI:30.02–44.24). High heterogeneity was observed with I^2^ of 99.4%, *P* < 0.000. According to subgroup analysis, the pooled prevalence was high in the SNNPR (41.49%: 95%CI: 19.52–63.46) followed by the Amhara region (41.11%: 95%CI: 30.41–51.8), the Tigray region (31.40%: 95%CI:11.72–51.09), and the Oromia region (28.98%: 95%CI: 18.85–39.1). Year from 2011 to 2015 contributed to the highest prevalence of *Schistosoma mansoni* infection among children (46.31%: 95%:34.21–59.05).

**Conclusion:**

This study revealed a 37.13% prevalence of *Schistosoma mansoni* infection among children. This is an alert to improve and implement appropriate control strategies such as mass drug administration in Ethiopia.

## Introduction

Schistosomiasis is a neglected tropical disease caused by blood-dwelling digenetic trematodes of the genus *Schistosoma*. It affects mainly the poor and marginalized segment of the world [1]. Schistosomiasis affects more than 240 million people and leads to the loss of 70 million disability-adjusted life years globally. About 800 million people are at risk of infection in 78 countries [2]. Schistosomiasis imposes a negative impact on child development, pregnancy outcome, and agricultural productivity [3]. Children are the more risky group with high morbidity and mortality, in addition, the disease results in anemia, malnutrition, decreased aerobic capacity, growth delay, cognitive and memory impairment [4].

Approximately 207 million people required preventive treatment for Schistosomiasis in 2016 worldwide. Different global and national disease monitoring and control initiatives are ongoing to control Schistosomiasis [5, 6]. These programs are mainly based on mass drug administration (MDA) with praziquantel. Achievement is evaluated base on the successful treatment of 75% of the school-age children (SAC) [7, 8].

In Ethiopia, Schistosomiasis is a major public health problem caused by *Schistosoma mansoni* and *Schistosoma hematobium* with 5.1 million people infected and 37.3 million people being at risk of infection. Of these, 3.4 million, 12.3 million, and 21.6 million are pre-school children, school-aged children, and adults respectively [9, 10]. The pooled prevalence of *S. mansoni* infection in Ethiopia was reported to be 18.7% (95%CI: 14.7–23.5) [11]. Another meta-analysis showed the prevalence of *S. mansoni* and *S. hematobium* among school-age children to be 28.78% (95% CI: 23.81, 33.74) [12]. The prevalence of *S. mansoni* among children ranges from 4.9 to 89.9% [13, 14]. A population-based survey conducted in the Amhara region, Northern Ethiopia involving 15,455 children (6 to 15 years old) reported a 6.9% regional prevalence of *S. mansoni* [15]. National mapping of soil-transmitted helminths and *S. mansoni* in Ethiopia assessed the prevalence of soil-transmitted helminths and *S. mansoni* from 2650 children of 9–14 years old in the Amhara region and found a 2.6% prevalence of *S. mansoni* [16].

Different prevalence studies targeting School-age children had been also conducted in different parties of the country. In the Southwest part of Ethiopia; in Mizan-Aman town 44.8% [17], in Jimma town 28.7% [18], and Ejaji town in the West Shoa Zone (12.94%) [19]. In the Northwest part of Ethiopia; Lake Tana 14.3% [20], Western Tigray 26.3% [21], Zarima town 37.9% [22], Mekelle city 23.9% [23], Waja Timuga 73.9% [24], rural Bahir Dar 47.2% [25], Sanja town 82.8% [26], Haik town 45% [27], Maksegnit and Enfranz towns 49% [28], and in the of West port of Ethiopia; Fincha’a sugar estate 53.2% [29].

In Ethiopia, more than 14 million SAC require MDA [30]. The Federal Ministry of Health (FMOH) of Ethiopia mapped 346 Schistosomiasis endemic districts and implemented MDA through integrated training, drug distribution, mobilization, technical staff involvement, and resource mobilization. In 2016 about 2.5 million SAC were targeted for MDA and 1.86 million were treated, this showed 74.4% effectiveness of the MDA program [9]. To control and eliminate Schistosomiasis in Ethiopia, providing a national view of the disease prevalence, and identifying endemic areas are highly significant. Indicating the prevalence of *Schistosoma mansoni* among children at the national and regional level is a valuable asset for designing the type of prevention and control strategy and implementation of the MDA program. It is also important to indicate the endemic areas and to know the current status of Schistosomiasis in children.

## Materials and methods

### Search strategy and selection criteria

A systematic search of potentially eligible studies was carried out from September 01/2020 to November 31/2020 in PubMed, Google Scholar, Web of Science, Scopus, ResearchGate, MEDLINE, and Google. An additional search was made by June 2021. The literature search was carried out using the search term “((prevalence or magnitude or epidemiology) and (*Schistosoma mansoni* or intestinal schistosomiasis or Schistosomiasis) and (pre-school children or school-age children or children) and (Ethiopia))”. Articles published from the proceedings of professional associations such as the Ethiopian Medical Laboratory Association, the Ethiopian Public Health Association, and the annual national research conference were searched. The reference lists of the retrieved studies and reviews were also searched for additional articles.

The included studies were identified after two reviewers independently screened the title, abstract, and the full-text of the articles obtained from the search, and the results were cross-checked by the third reviewer. The final selection was based on the full-text evaluation.

### Data extraction and quality assessment

The outcome variable for this study was the prevalence of *S. mansoni* infection among children of 6 months to 19 years old in Ethiopia. Data were extracted by the two reviewers independently using a Microsoft excel extraction sheet. The sheet contains information including the name of the primary author, year of publication, study design, diagnostic methods, study group, sample size, number of male participants, and number of female participants, number of positive cases, the prevalence of *S. mansoni,* and the region where the study was conducted.

The quality of each study was evaluated following the Joanna Briggs Institute (JBI) critical appraisal checklist for prevalence studies [31]. Studies were assessed according to the appropriateness of the method used, validity and accuracy of the diagnostic methods, adequacy of the sample size, validity of the sampling procedures, appropriateness of the study design, and the statistical analysis. Each selected study was assessed using 10 quality control items and for each fulfilled item, a score of 1 was given while 0 was given for each of the unfulfilled items. An aggregate of all the scores was generated and converted into an index. Based on the quality indices generated, studies were classified as having low (0.0–0.3), moderate (0.4–0.6), or high (0.7–1.0) quality.

### Eligibility criteria

#### Inclusion criteria

The reviewers carefully screened the title, abstract, and full text of each published article for its relevance, and eligibility. Original studies reporting the prevalence of *S. mansoni* infection among children in Ethiopia were included in this systematic review and meta-analysis.

#### Exclusion criteria

Studies were excluded if they were reported in a language other than English, used inappropriate study design, and non-representative sample size. Studies conducted in a selective population, and not included relevant extractable data were excluded. Case reports, reviews, and studies conducted on adults were also excluded.

#### Data synthesis and statistical analysis

The pooled prevalence was calculated by using the Metan commands in STATA version 12. The pooled effect size was presented in the form of a forest plot. To account for the studies variability, the meta-analysis was carried out using the random effect model. The amount of between-studies heterogeneity was quantified using I^2^ statistics, which describes the proportion of total variation of the effect estimates resulting from the between-studies heterogeneity and values can be from 0 to 100%. The I^2^ values of 25, 50, and 75% were considered low, moderate, and high heterogeneity, respectively [32].

The potential influence of the covariates on the pooled effect estimate was investigated by subgroup analysis. Subgroup analysis was done by the region where the studies were conducted, year of publication, sample size, and sex of study participants. Publication bias was assessed by visual inspection of symmetry of the funnel plot and the egger’s test statistics. *P*-value ≤0.05 and asymmetry of the funnel plot indicate the presence of publication bias [33].

## Result

### Selection and identification of studies

**Fig. 1:**
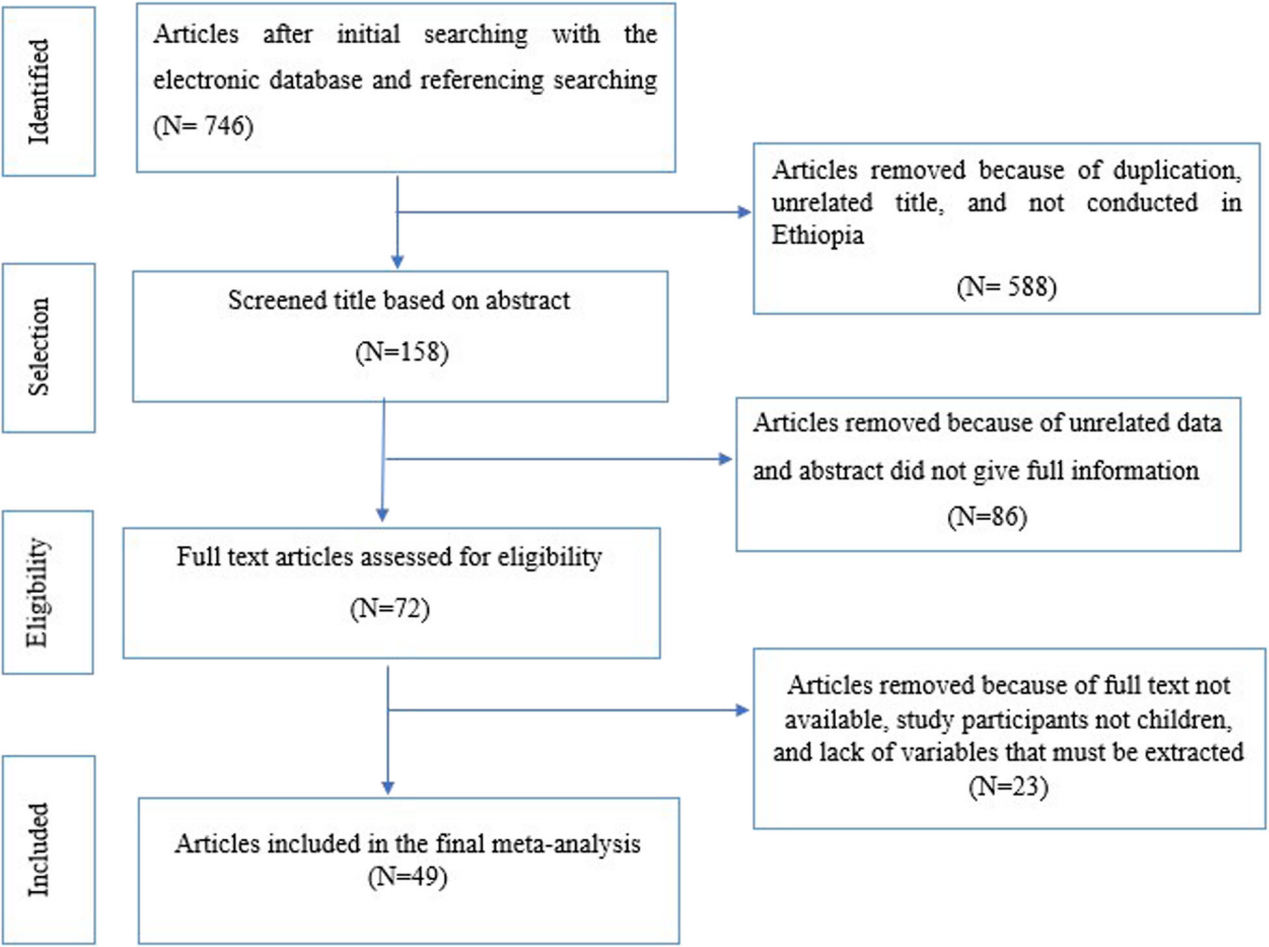
Flow chart of selection of studies

A total of 746 articles were identified through online searching and reference screening. After the initial screening of the title and abstract of the identified studies, the full text of potentially eligible studies was retrieved for detailed assessment. A total of 674 articles were ineligible and excluded. The remaining 72 articles were assessed in detail. About 24 studies were excluded since they lack variables that must be extracted for analysis leaving 49 potentially eligible studies for the final meta-analysis. Preferred Reporting Items for Systematic Review and Meta-analysis statement (PRISMA checklist 2009) was followed [34] (Fig. 1).

### Characteristics of included studies

Forty-nine studies involving 20,493 children (10,572 male and 9, 921 females) were included in this meta-analysis. The age of the study participants ranged from 6 months to 19 years old. The studies were conducted from 2001 to 2021 in five national regional states of Ethiopia. Twenty-five studies were conducted in the Amhara region, 11 studies in the Oromia region, 7 studies in the Southern Nation Nationalities and People Region (SNNPR), 5 studies in the Tigray region, and 1 study in the Somali region of Ethiopia. All the included studies had a cross-sectional design and estimated point prevalence. The sample size of the studies ranged from 233 to 798. The majority of the studies used Kato Katz thick smear as a diagnostic method and one study use circulating cathodic antigen (CCA) test (Table 1).

**Table 1:** Characteristics of included studies

Author/reference	Year	Region	Sample size	Diagnostic methods	Prevalence (%)
Tiruneh A et al [22]	2020	SNNPR	389	Kato-Katz thick smear	19.3
Alemu A et al [23]	2011	Amhara	319	Kato-Katz thick smear	37.9
Alemu A et al [24]	2016	Amhara	401	Kato-Katz thick smear	11.2
Assefa A et al [25]	2013	Tigray	457	Kato-Katz thick smear	23.9
Tefera A et al [18]	2020	Oromia	328	Kato-Katz thick smear	28.7
Tadege B et al [35]	2017	SNNPR	384	Kato-Katz thick smear	31
Alemayehu B et al [36]	2015	SNNPR	384	Kato-Katz thick smear	81.3
Alemayehu B et al [37]	2017	SNNPR	503	Kato-Katz thick smear	58.6
Mathewos B et al [38]	2014	Amhara	261	Kato-Katz thick smear	33.7
Feleke D et al [39]	2017	Amhara	279	Formol-Ether concentration and direct wet mount	80.5
Gashaw F et al [28]	2015	Amhara	550	Kato-Katz thick smear	49
Amsalu G et al [27]	2015	Amhara	380	Kato-Katz thick smear	45
Alebie G et al [26]	2014	Amhara	384	Kato-Katz thick smear	82.8
Desta H et al [40]	2014	Tigray	469	Kato-Katz thick smear	42.4
Wubet K et al [41]	2020	Amhara	362	Formol-Ether concentration	15.2
Workineh L et al [42]	2019	Amhara	422	Kato-Katz thick smear	24.9
Worku L et al [14]	2014	Amhara	385	Kato-Katz thick smear and Formol-Ether concentration	89.9
Alemu M et al [43]	2014	SNNPR	405	Kato-Katz thick smear	12.6
Bajiro M et al [44]	2017	Oromia	500	Kato-Katz thick smear	27.6
Bajiro M et al [45]	2018	Oromia	233	Kato-Katz thick smear	26.6
Ansha M et al [46]	2020	Oromia	298	Kato-Katz thick smear	11.4
Hailu T et al [25]	2018	Amhara	409	Ritchie’s concentration	13.7
Ibrahim T et al [19]	2018	Oromia	340	Kato-Katz thick smear	12.94
Bekana T et al [47]	2019	Oromia	317	Kato-Katz thick smear	42.9
Teshale T et al [13]	2018	Tigray	410	Kato-Katz thick smear	4.9
Teklemariam A et al [48]	2018	Tigray	480	Formol-ether concentration	23.13
Fentie T et al [49]	2013	Amhara	520	Kato-Katz thick smear	16.7
Mitiku H et al [50]	2010	Oromia	375	Kato-Katz thick smear	12
Legesse L et al [51]	2010	Tigray	381	Kato-Katz thick smear and Formol-Ether concentration	63
Jemaneh L et al [52]	2001	Amhara	687	Kato-Katz thick smear	19.4
Endris M et al [35]	2010	Amhara	354	Kato-Katz thick smear	43.5
Essa T *el al* [36]	2013	Amhara	579	Kato-Katz thick smear	20.6
Addisu T et al [37]	2015	Amhara	365	Kato-Katz thick smear	15.9
Tulu B et al [53]	2014	Oromia	340	Formol-Ether concentration	12.6
Kemal M et al [39]	2019	Somali	236	Kato-Katz thick smear	25
Bajiro M et al [54]	2016	Oromia	500	Kato-Katz thick smear	24
Reta B et al [55]	2013	Amhara	342	Kato-Katz thick smear	70.47
Erko B et al [56]	2012	SNNPR	299	Kato-Katz thick smear	74.9
Haile S et al [43]	2012	Oromia	324	Kato-Katz thick smear	67.6
Woldegerima E et al [44]	2018	Amhara	372	Kato-Katz thick smear	35
Tesfie A et al [45]	2020	Amhara	245	Kato-Katz thick smear	83.3
Abdi M et al [57]	2016	Amhara	408	Formol-Ether concentration	29.9
Bekana T et al [58]	2021	Amhara	798	Kato-Katz thick smear and Formol-Ether concentration	25.6
Mekonnen Z et al [29]	2014	Oromia	453	Kato-Katz thick smear	53.2
Jejaw A et al [17]	2015	Amhara	460	Kato-Katz thick smear and Formol-Ether concentration	44.8
Degarege A et al [59]	2016	Amhara	403	Kato-Katz thick smear and Formol-Ether concentration	24.6
Degarege A et al [60]	2014	Amhara	620	Triple urine-CCA-cassette	81.1
Zeleke A et al [61]	2020	Amhara	786	Kato-Katz thick smear	33.5
Shumbej T et al [62]	2019	SNNPR	597	Kato-Katz thick smear	12.9

### Prevalence of *Schistosoma mansoni* among children in Ethiopia*,* 2001–2021

**Fig. 2:**
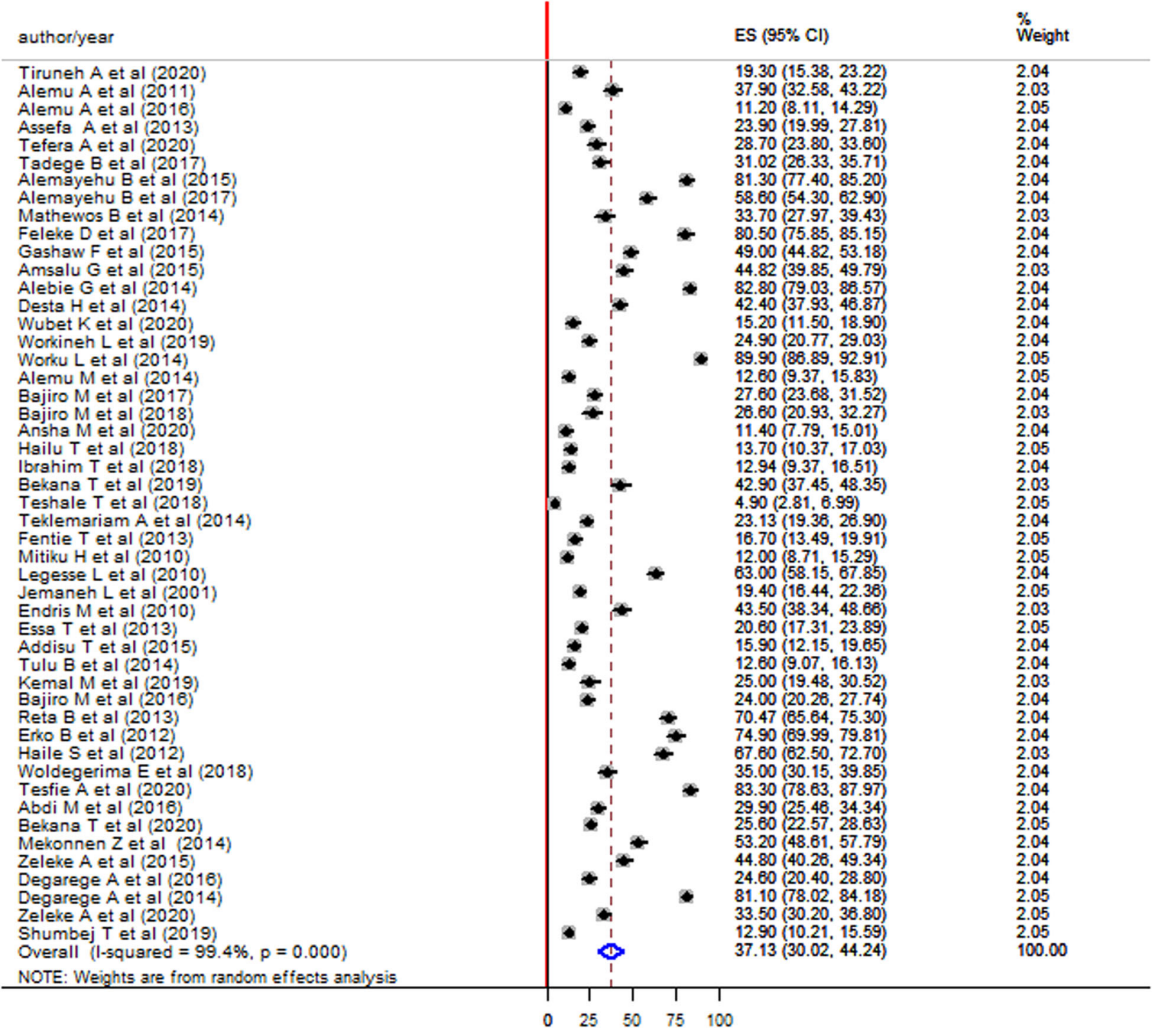
Forest plot showing the pooled prevalence of *S. mansoni* infection among children in Ethiopia 2001–2021

The prevalence of *S. mansoni* infection among children in the included studies was ranged from 4.9% reported in the Tigray region by Teshale T. et al, 2018 [13] to 89.9% reported from the Amhara region by Worku L. et al 2014 [14]. This meta-analysis and systematic review showed 37.13% (95% CI:30.02–44.24) pooled prevalence of *S. mansoni* infection among children using the random effect model. High heterogeneity was observed across the included studies with an I^2^ value of 99.4%, *p*-value< 0.000 (Fig. 2).

### Subgroup analysis

**Fig. 3:**
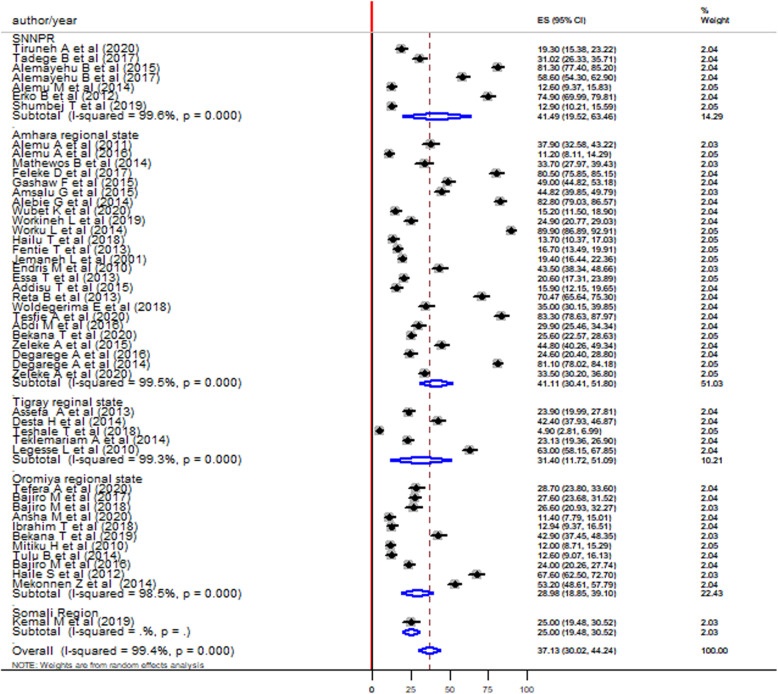
Forest plot showing the pooled prevalence of *S. mansoni* infection among children by the regions of Ethiopia

Subgroup analysis was conducted by the region where the studies were conducted, publication year, categorized sample size, and sex. According to the region, the highest pooled prevalence of *S. mansoni* infection among children was in the SNNPR (41.49, 95%CI: 19.52–63.46) followed by the Amhara region (41.11, 95%CI: 30.41–51.8), the Tigray region (31.40, 95%CI: 11.72–51.09) and the Oromia region (28.98, 95%CI: 18.85–39.10) (Fig. 3).

**Fig. 4:**
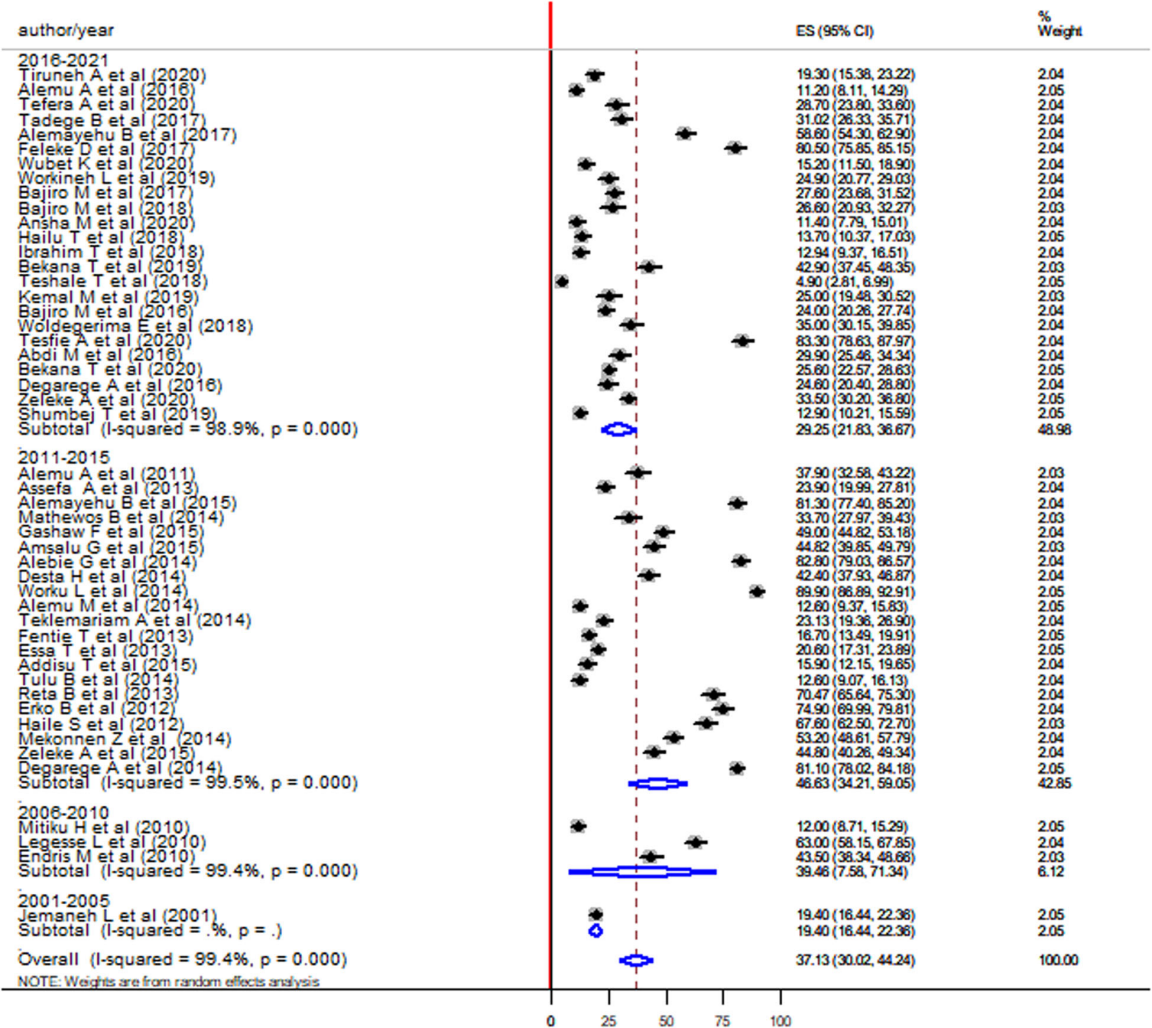
Forest plot showing the pooled prevalence of *S. mansoni* infection among children in Ethiopia by year of publication

According to the publication year, the highest pooled prevalence of *S. mansoni* infection was observed in studies conducted from 2011 to 2015 (46.31, 95%CI:34.21–59.05) followed by 2006 to 2010 (39.46, 95%CI: 7.58–71.34) and 2016 to 2021 (29.25, 95%CI: 24.41–38.70) (Fig. 4).

**Fig. 5:**
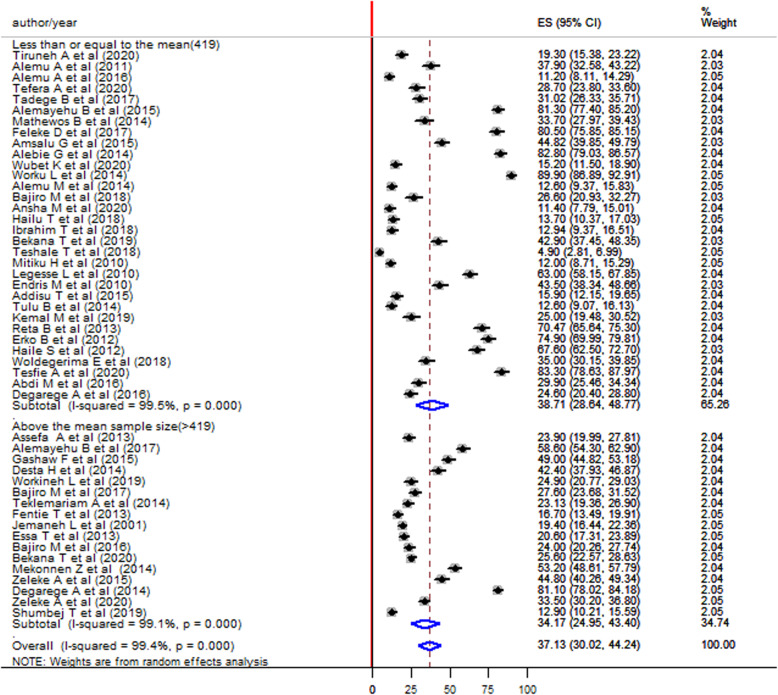
Forest plot showing the pooled prevalence of *S. mansoni* infection among children in Ethiopia by sample size

**Fig. 6:**
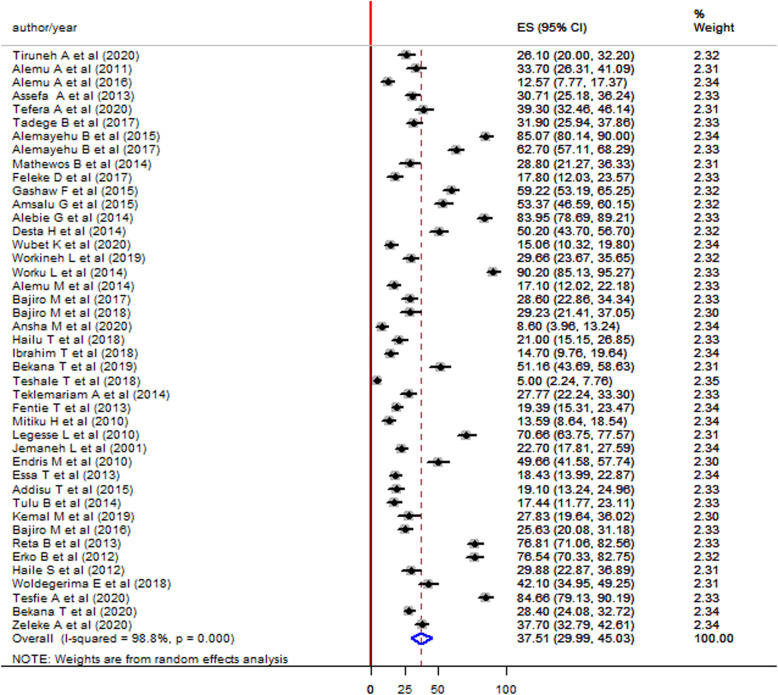
Forest plot showing the pooled prevalence of *S. mansoni* infection in males

**Fig. 7:**
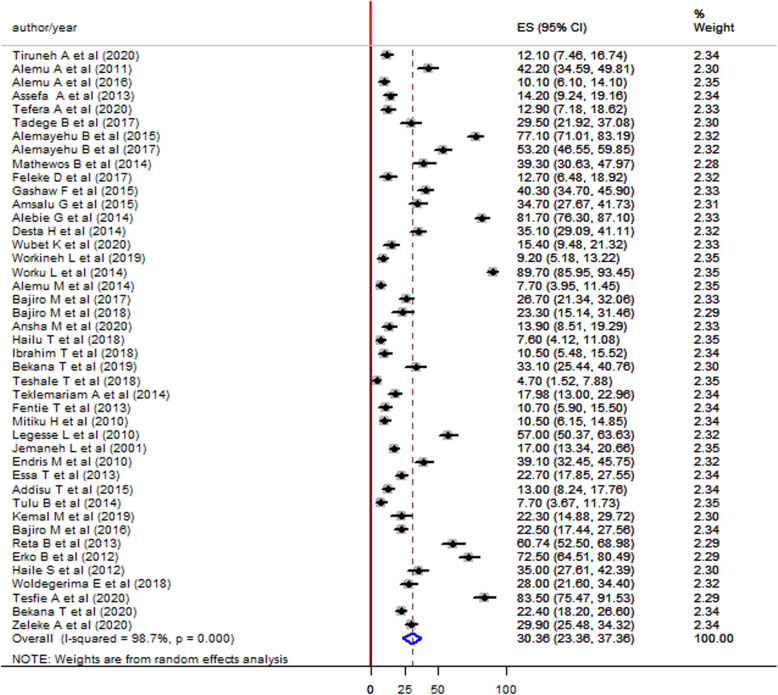
Forest plot showing the pooled prevalence of *S. mansoni* infection in females

The pooled prevalence was higher among studies that have a sample size of less than or equal to the average sample size (≤419) compared to those that have a sample size of greater than the average sample size (> 419) (38.71% vs 34.17%) (Fig. 5). The pooled prevalence was higher in males (37.51, 95%CI: 29.99–45.03) (Fig. 6) compared to females (30.36, 95%CI, 23.36–37.36) (Fig. 7).

### Publication bias

**Fig. 8:**
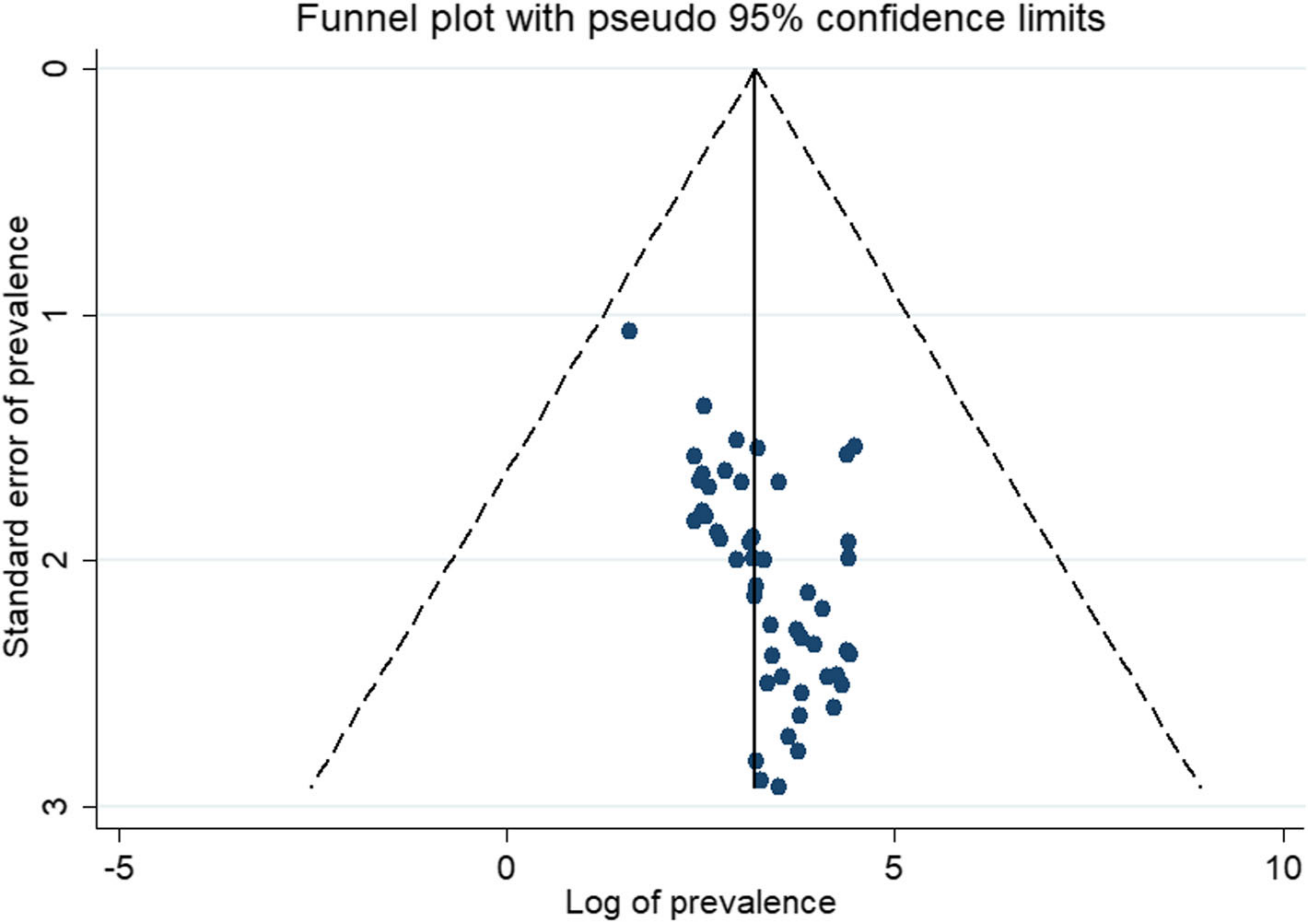
Funnel plot

The publication bias was assessed by visual inspection of the funnel plot (Fig. 8) and using egger’s test statistics. The logit of proportion and its standard error were used to evaluate the presence or absence of bias. The result showed the absence of publication bias with a *p*-value of 0.320.

### Sensitivity analysis

Sensitivity analysis was done to check the impact of a single study on pooled prevalence. All the studies were omitted sequentially and the pooled effect size in all cases was within the 95% confidence interval of the combined effect size. This indicates the absence of a single study impact on the pooled effect size (Table 2).

**Table 2:** Sensitivity analysis

Study omitted	Estimate	95% Confidence interval
Tiruneh A et al (2020) [51]	37.50	30.27–44.74
Alemu A et al (2011) [22]	37.12	29.89–44.34
Alemu A et al (2016) [52]	37.67	30.46–44.88
Assefa A et al (2013) [23]	37.41	30.16–44.65
Tefera A et al (2020) [18]	37.31	30.08–44.54
Tadege B et al (2017) [35]	37.26	30.02–44.49
Alemayehu B et al (2015)	36.21	29.23–43.19
Alemayehu B et al (2017) [36]	36.68	29.50–43.87
Mathewos B et al (2014) [37]	37.20	29.99–44.42
Feleke D et al (2017) [39]	36.23	29.18–43.28
Gashaw F et al (2015) [28]	36.88	29.66–44.11
Amsalu G et al (2015) [27]	36.97	29.75–44.19
Alebie G et al (2014) [26]	36.18	29.23–43.13
Desta H et al (2014) [40]	37.02	29.79–44.25
Wubet K et al (2020) [41]	37.59	30.36–44.81
Workineh L et al (2019) [42]	37.39	30.14–44.63
Worku L et al (2014) [14]	36.02	29.39–42.66
Alemu M et al (2014) [43]	37.64	30.42–44.86
Bajiro M et al (2017) [44]	37.33	30.08–44.58
Bajiro M et al (2018) [45]	37.35	30.13–44.57
Ansha M et al (2020) [46]	37.67	30.46–44.87
Hailu T et al (2018) [25]	37.62	30.4–44.85
Ibrahim T et al (2018) [19]	37.64	30.42–44.85
Bekana T et al (2019) [47]	37.01	29.8–44.23
Teshale T et al (2018) [13]	37.81	30.72–44.89
Teklemariam A et al (2018) [48]	37.42	30.17–44.67
Fentie T et al (2013) [63]	37.56	30.31–44.80
Mitiku H et al (2010) [64]	37.66	30.44–44.87
Legesse L et al (2010) [65]	36.59	29.43–43.76
Jemaneh L et al (2001) [66]	37.50	30.23–44.78
Endris M et al (2010) [67]	37.0	29.78–44.23
Essa T et al (2013) [68]	37.48	30.22–44.74
Addisu T et al (2015) [69]	37.57	30.35–44.80
Tulu B et al (2014) [53]	37.64	30.43–44.86
Kemal M et al (2019) [70]	37.38	30.17–44.6
Bajiro M et al (2016) [54]	37.41	30.15–44.66
Reta B et al (2013) [55]	36.44	29.31–43.56
Erko B et al (2012) [56]	36.35	29.24–43.45
Haile S et al (2012) [71]	36.5	29.35–43.64
Woldegerima E et al (2018) [72]	37.18	29.94–44.41
Tesfie A et al (2020) [73]	36.17	29.14–43.20
Abdi M et al (2016) [57]	37.28	30.04–44.52
Bekana T et al (2020) [58]	37.37	30.1–44.67
Mekonnen Z et al (2014) [29]	36.79	29.59–44.01
Jejaw A et al (2015) [17]	37.01	29.75–44.21
Degarege A et al (2016) [59]	37.39	30.152–44.63
Degarege A et al (2014) [60]	36.21	29.36–43.06
Zeleke A et al (2020) [61]	37.21	29.92–44.49
Shumbej T et al (2019) [62]	37.64	30.40–44.87
**Combined**	**37.13**	**30.02–44.24**

## Discussion

*Schistosoma mansoni* infection continues to be a major public health problem worldwide with 240 million cases [2]. To our knowledge, this study is the first comprehensive systematic review and meta-analysis of primary studies to indicate the prevalence of *S. mansoni* infection among children at the national level. We performed compressive reviews and meta-analyses of 49 cross-sectional studies reporting the prevalence of *S. mansoni* infection among children in Ethiopia. Ethiopia is continuously implementing the MDA program with large-scale periodic treatment since 2010 to reduce the burden of Schistosomiasis and other intestinal parasitic infections. The country has also planned to reduce the national prevalence of Schistosomiasis below 1 % [16].

This systematic review and meta-analysis showed the pooled prevalence of *S. mansoni* infection among children as 37.13% (95%CI: 30.02–44.24). This result indicates as Schistosomiasis is still a major public health problem among children in Ethiopia. This finding was higher than the previous meta-analysis conducted in Ethiopia by Hussen et al., and Woldeyohannes et al. that reported 10.9 and 28.78% pooled prevalence of *S. mansoni* among children respectively [11, 12]. It was also higher than the reports from Mozambique (8.7%) by Augusto et al., Sierra Leone (16.3, 18.4%) by Sesay et al.*,* Koroma et al., Zimbabwe (7.2%) by Midzi et al., and Uganda (25.6%) by Exum et al. [74–78]. The difference might be due to geographical and ecological variation, the difference in implementation of the Schistosomiasis control programs, the difference in the sample size, and the difference in diagnostic methods. The difference in safe water supply and sanitation practices in schools might also have a role in the variability of the prevalence since they do have a vital role in the reduction of Schistosomiasis [79]. The habit of open field defecation and the use of untreated night soil as fertilizer can also increase the transmission of Schistosomiasis [78].

High between-study heterogeneity was noted with an I^2^ value of 99.4%. To account for this heterogeneity, subgroup analysis was done according to sex, sample size, year of publication, and regions where the studies were conducted. However, the heterogeneity remains high after subgroup analysis.

According to subgroup analysis, the pooled prevalence of *S. mansoni* infection was found to be higher among males than females (37.51% vs 30.36%). This was supported by previous studies in Ethiopia that reported being male is 58.3% more likely to be infected with Schistosomiasis than females and in Brazil [12, 80]. The high prevalence in males might be due to bathing in open freshwater bodies of males than females, outdoor activities of male children in rural areas than females, and engagement of males in irrigation and agricultural activities [58].

The pooled prevalence of *S. mansoni* infection among children was highest in the SNNPR 41.49% (95%CI: 19.52–63.46) followed by the Amhara region 41.11%, (95%CI: 30.41–51.80), the Tigray region 31.4%, (95%CI: 11.72–51.09), and the Oromia regional state 28.98%, (95%CI: 18.85–39.10). This variation might be due to the different environmental and ecological variations, the difference in population density, and variability in efficacy of praziquantel [81–83]. The difference in environmental sanitation, personal hygiene, altitude differences, sanitation of streams/rivers, ponds, epidemiology of the snail vector, habit of open defecation, utilization of night soil as a fertilized might also contribute to the difference [84]. The other explanation is that the SNNPR is rich in water bodies which can be favorable for the intermediate snail species.

The current prevalence in the Amhara region (41.11%) is higher than previous studies that reported the regional prevalence of *S. mansoni* as 6.9 and 2.6% [15, 16]. The variation might be due to differences in sample size, diagnostic methods, and different levels of endemicity in Schistosomiasis. Most of the studies included in this meta-analysis are conducted in Schistosomiasis endemic areas while the previous studies included children from different schools in the region irrespective of endemicity of Schistosomiasis.

The pooled prevalence was found to be high in studies conducted 2011 to 2015 (44.46, 95%: CI: 30.88–58.03) followed by 2006 to 2010 (39.46, 95%CI: 7.58–71.34) and 2016 to 2020 (32.09 95%CI: 22.84–41.34). The reduced prevalence from 2016 to 2020 might be due to the beginning of the mass drug administration (MDA) program in Ethiopia by 2015 [85]. Implementation, monitoring, and evaluation of the Schistosomiasis and Soil-transmitted helminths control program such as mass drug administration program in school [9]. One national survey conducted in 2019 had reported 75.5% coverage of Praziquantel (PZQ) treatment against school children, this might play a role in the reduction of *S. mansoni* prevalence among school-age children in 2016–2021 [49].

The studies were grouped as studies having above and below the average sample size (the average sample size was 419). The pooled prevalence was 43.93%, (95%CI: 32.47–55.40) in studies with a sample size of ≤419 and 27.01% (95%CI: 20.47–33.56) in studies with sample size > 419. The between studies heterogeneity remained high even after subgroups analysis.

The symmetry of the funnel plot and the statistical analysis with the egger’s test statistics ruled out the absence of publication bias with a *p*-value of 0.320.

Our study has limitations such as; between-study heterogeneity among enrolled studies was high which might be due to variations in study designs, methodologies, sample populations, and methods of diagnosis employed by the various studies. Only articles published in English were included in the meta-analysis. We did not review the factors that are associated with the prevalence of *S. mansoni* infection. The studies were not evenly distributed across different regions of Ethiopia. The majority of studies included in this systematic review and meta-analysis used Kato-Katz diagnostic method, which is less sensitive than circulating cathodic antigen (CCA), and polymerase chain reaction (PCR). The Kato-Katz test is less sensitive and the egg can be missed due to different reasons like the presence of the egg in the unexamined part of the stool and excretion of the egg by another day this leads to underestimation of the prevalence of *S. mansoni* infection [50].

## Conclusion and recommendation

This systematic review and meta-analysis summarized the high prevalence of *S. mansoni* infection among children in Ethiopia. This shows that *S. mansoni* is still a major public health problem in Ethiopia. The pooled prevalence of *S. mansoni* infection is highest in the SNNPR. Males were more affected than females. This systematic review and meta-analysis identified a wide range of the prevalence of *S. mansoni* among children in Ethiopia from 2001 to 2021.

Finally, our recommendation to the Ethiopian Federal Ministry of Health is to work with different stakeholders and recognize *S. mansoni* infection as still being a major health problem in the country, mainly among children, design long term Schistosomiasis control programs that should be achieved through the provision of safe drinking water in schools, establishing health education programs in schools and the community about personal hygiene, environmental hygiene, proper disposal of waste, keeping water bodies clean, and establishment of sanitation campaigns in each school and community. In addition, increasing the geographical coverage of MDA and improving the delivery of the drug to the MDA sites are also crucial.

Further studies that more sensitive techniques like CAA, PCR should be conducted in endemic areas to indicated the correct estimate. In addition, studies that assess the efficacy of praziquantel periodically and reinfection rate should be motivated.

## Data Availability

All the necessary materials are available within the manuscript.

## Abbreviations

CCA: Circulating cathodic antigen
MDA: Mass drug administration
PCR: Polymerase chain reaction
SAC: School age children
SNNPR: Southern Nation Nationalities and People’s Region
